# Does the Position of the Polyaxial Screw Head in Patients With L5-S1 Stabilization Lead to an Increased Difficulty in L5 Transforaminal Nerve Injection? A Three-Dimensional Computerized Tomography Study

**DOI:** 10.7759/cureus.14491

**Published:** 2021-04-14

**Authors:** Ilker Gulec, Feyza Karagoz Guzey

**Affiliations:** 1 Department of Neurosurgery, Bagcilar Training and Research Hospital, Health Sciences University, Istanbul, TUR

**Keywords:** transforaminal nerve injection, l5-s1 level, stabilization, three-dimensional ct

## Abstract

Background

Challenges may be encountered if transforaminal nerve injection (TFNI) is required in patients who have undergone posterior transpedicular stabilization (PTS) surgery to the L5-S1 level. In this study, we investigated the contributory factors that lead to these challenges.

Methods

We selected 125 patients who underwent PTS surgery involving the L5-S1 segment, between 18 to 70 years of age to be included in the study. The demographic data of the patients, body mass indexes (BMI), postoperative spondylolisthesis grades, heights of the iliac crest, and the positions of the polyaxial screw head were assessed. The shortest trajectory of L5-TFNI, the distance of the needle entry point (NEP) to the midline, and optimum viewing angles (VA) were measured on the three-dimensional computed tomography (CT) sections.

Results

Pre-PTS surgery, in males compared to females, NEP was noted to be more medial (p=0.007), the needle trajectory was shorter (p=0.001), and the optimal VA was narrower (p=0.001). Increasing BMI and increasing height of the iliac crest caused the TFNI trajectory to become longer. Post-PTS surgery, angulation of polyaxial screw heads of more than 15 degrees laterally in both genders significantly caused a decrease in VA (p=0.001).

Conclusions

Using the reconstruction technique in 3D CT, we demonstrated that pedicle screw heads angled laterally, a higher iliac crest height, and an increased BMI make L5-TFNI difficult to be performed. Locking the stabilization system while targeting the most neutral position for polyaxial screw heads during surgery may facilitate the L5-TFNI.

## Introduction

Radiculopathy is frequently encountered in patients who have undergone posterior transpedicular stabilization (PTS) surgery [1-4]. Anamnesis, neurological examination, electrodiagnostic tests, and magnetic resonance imagination are often used to diagnose the root generated pain [2,5,6]. However, the above-mentioned diagnostic modalities are sometimes insufficient for diagnosis because of the patients' advanced age and some degree of ongoing lumbar degenerative changes already present in these patients [5,7]. Nowadays, the transforaminal nerve injection (TFNI), which had an accuracy rate of 68%-91.8% [2,5], is recommended for diagnosing the root causing radiculopathy [3]. In addition to this, transforaminal (TF) injections successfully provided adequate pain control for approximately three months in patients [6] and also decreased the need for opioid prescribing [1,8] and the need for surgery [8,9].

Transforaminal interventions to the L5-S1 level are quite challenging in the lumbar region [3,4,10-12]. Congenital differences of the spine such as scoliosis [5,13], high iliac crest [3,4,10-12], large transverse process and sacral ala [7], or degenerative changes in the spine such as increased lumbar lordosis [11,14], stenosis of the neural foramen [7,10] and facet hypertrophy [7,10] are the major factors that make this approach difficult to be performed. The difficulty increases, even more, when L5-TFNI is required after PTS surgery performed at L5-S1 levels, because the implant components cause the viewing angle (VA) for L5-TFNI to be narrowed or blocked posterior aspect. We searched the literature and found no study evaluating L5-TFNI in patients with PTS surgery involving the L5-S1 segment.

In lumbar spinal interventions, the guidance of C-arm X-ray devices is frequently preferred [2,5,12]. During the procedure, the angle of view of the device must be adjusted because of the superimposed images of the anatomical structures [12,13]. Sometimes, the complications such as improper or intrathecal placement of the needle and spinal cord damage may occur [5]. In addition to that, multiple attempts to place the needle at the target point reduce the comfort of the patients and expose them to high radiation [14]. Those difficulties in performing L5-TFNI in patients with PTS can be overcome by using three-dimensional (3D) computerized tomography (CT). It allows high-resolution and 3D image reconstructions to be produced and interventions with higher accuracy in a short time while minimizing the risk of complications [14,15]. It also has the advantage of simulating the entire procedure [15]. 

So, we investigated whether the body mass index (BMI), the degree of spondylolisthesis, the height of the iliac crest, and the spatial location of the parts of the PTS implant make the L5-TFNI procedure difficult to be performed using 3D CT simulation method.

## Materials and methods

This retrospective study was approved by the local ethics committee (2020.07.1.05.098). Records of 149 patients between 18 to 70 years of age who underwent PTS surgery involving L5-S1 levels in our clinic between 2015 and 2020 were analyzed. One hundred and twenty-five patients who met the inclusion criteria were included in the study. The demographic data of the patients, the BMI, the grades of spondylolisthesis after PTS surgery, the heights of the iliac crests, and the spatial positions of the screw heads (SH) of the stabilization implants of the patients were determined. Assuming these patients had L5 sciatalgia, measurements were made on 3D CT sections before and after PTS surgery by simulating the L5-TFNI procedure.

Inclusion criteria

1. Patients between 18 to 70 years of age, 2. Patients who underwent PTS surgery using a polyaxial pedicle screw including lumbar L5-S1 levels, and 3. Patients with preoperative and postoperative lumbar CT with a slice thickness of 1.5 mm or thinner.

Exclusion criteria

1. Patients with a diagnosis of trauma, tumour, infection in the lumbar spine, congenital spinal anomaly or scoliosis, 2. Patients who underwent posterior lumbar interbody implantation with PTS surgery, and 3. Patients with sections of lumbar CT images thicker than 1.5 mm or having poor image quality.

Description of the measurements

**Figure 1:**
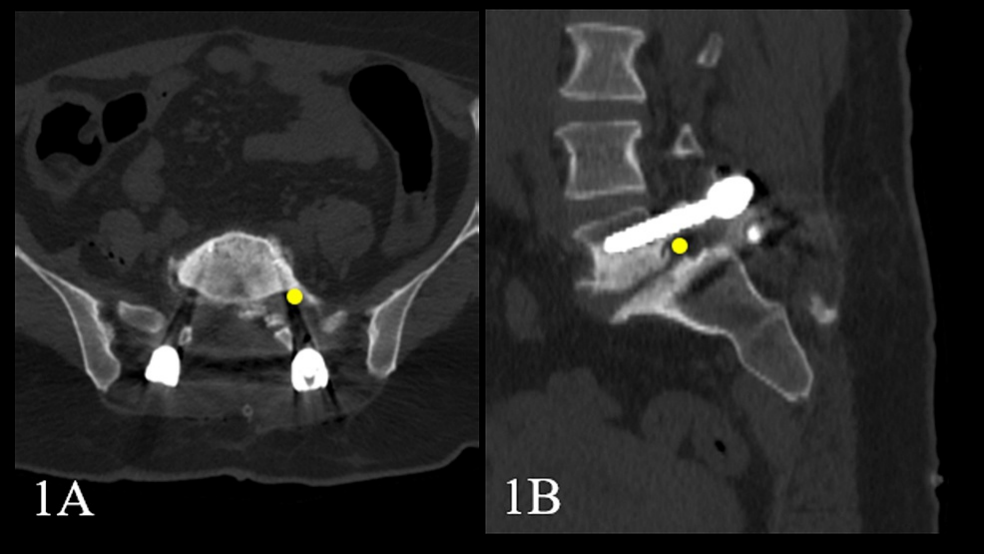
The lumbar 3D CT images demonstrate the target point where the needle tip is planned to be placed to perform L5 transforaminal nerve injection (yellow dot) in (A) axial and (B) in sagittal planes.

1. *Classification of BMI*: According to their BMIs, females and males were divided into two groups: ≤29.9 kg/m² and ≥30 kg/m².

2. *Classification of the Height of the Iliac Crest*: As previously described [11], the dorsal highest point of the iliac crest was taken as a reference in lateral radiography. The patients were divided into three types: Type 1: If the reference point is below the lower border of L5 pedicle; Type 2: If the reference point is between the lower border of L5 pedicle; and Type 3: If the reference point is above the L5 superior endplate.

3. *Lumbar CT Imaging and 3D Processing*: The patients were positioned on a very thin mattress on the CT table in the supine position. The CT sections with a thickness of up to 1.5 mm were taken parallel to the vertebral endplates, covering between L1-S2 levels. These sections were converted into 3D using RadiAnt DICOM Viewer 2020.2 software (Medixant, Poznan, Poland).

4. *Description of Target Point for L5-TFNI Intervention*: For the L5 -TFNI intervention, the previously defined point was taken as the target (Figure 1) [5]. The subpedicular region of the L5 vertebra was divided into three vertical columns in the coronal plane. The point closest to the root in the outer column (A) was targeted by the tip of the needle.

**Figure 2:**
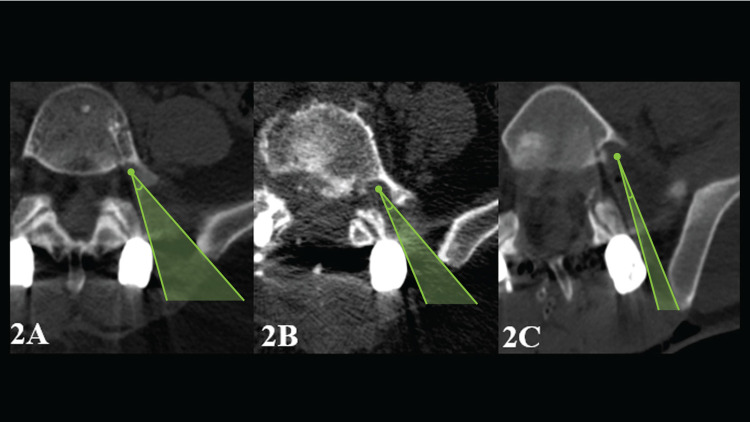
Positions of heads of the lumbar polyaxial screw were categorized as follows: (A) in the neutral (N) position (≤5˚); (B) LA-1 position, angulated laterally (between 5˚ and 15˚), and (C) LA-2 position, angulated more laterally (≥15˚). The figure also shows the measurement of the angle of view on axial images.

5.* Description of the Positions of the Polyaxial Screw Heads*: As shown in Figure 2, SHs were categorized as in the neutral (N) position (≤5˚); LA-1 position, angled laterally (between 5˚ and 15˚), and LA-2 position, angled laterally (≥15˚) in axial CT sections.

**Figure 3:**
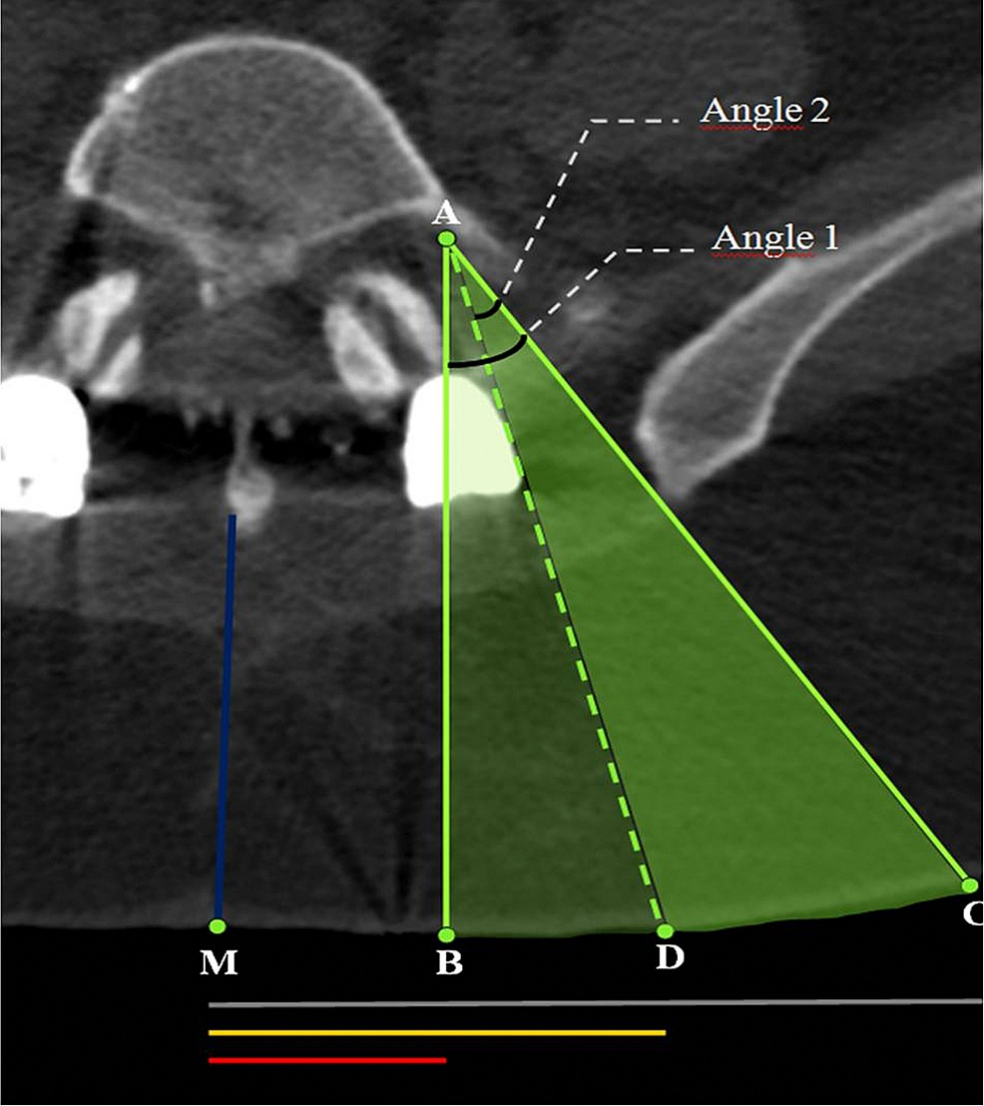
The measurements on axial lumbar computed tomography section. The lengths of the trajectories of A-B (shortest trajectory before transpedicular stabilization surgery), A-C, and A-D (shortest trajectory after transpedicular stabilization surgery) were measured. A, the target point for transforaminal nerve injection; B, The most medial entry point of the needle where transforaminal nerve injection can be performed before transpedicular stabilization surgery; C, The most lateral entry point of the needle where transforaminal nerve injection can be performed before transpedicular stabilization surgery; D, The most medial entry point of the needle where transforaminal nerve injection can be performed after transpedicular stabilization surgery; M: midline; Angle 1: the angle between the most medial and the most lateral trajectory where transforaminal nerve injection can be performed before transpedicular stabilization surgery; Angle 2:  the angle between the most medial and the most lateral trajectory where transforaminal nerve injection can be performed after transpedicular stabilization surgery. Gray, yellow, and red lines indicate the distance of the needle entry points from the midline.

6.* Measurements*: Measurements were made on the right and left sides of each patient, recorded as two different patients (Figure 3). Each measurement was made by an independent person who is an expert in the field and was repeated three times and averaged.

Statistics

The statistical analysis was made with NCSS (Number Cruncher Statistical System) 2007 Statistical Software (NCSS, LLC, Utah, USA) package program. In addition to descriptive statistical methods (mean, standard deviation), the distribution of variables was examined using the Shapiro-Wilk normality test. The paired t-test was performed for comparison of variables with normal distribution. A one-way analysis of variance for comparison of groups and a Tukey multiple comparison test was applied for comparison of subgroups. The independent t-test for the comparison of the independent groups, the Chi-square test for the comparison of qualitative data, and the Pearson correlation test for determining the relations of variables with each other were used. The results were evaluated at the level of significance p<0.05.

## Results

Demographic data and the results of the patients by gender are presented in Table 1.

**Table 1:** Patient demographics and results of the measurements by gender. SD, standard deviation; BMI, body mass index; ns, not significant; N, neutral (≤5˚);  LA-1 position, angled laterally (between 5˚and 15˚); LA-2 position, angled laterally (≥15˚); B, the most medial entry point of the needle where transforaminal nerve injection can be performed before transpedicular stabilization surgery; M, midline; D, needle entry point for the trajectory of transforaminal nerve injection passing just lateral to the screw head after transpedicular stabilization surgery;  C, the most medial entry point of the needle where transforaminal nerve injection allowed by the iliac crest can be performed before transpedicular stabilization surgery; A, the target point for transforaminal nerve injection. P<0.05 was considered statistically significant. The statistical significance levels were expressed as p<0.05, p<0.01, p<0.001. ns indicates p>0.05.

		Female (n=144)	Male (n=106)	p
Age, mean (± SD), year		56.4±12.4	50.3±14.8	<0.001
BMI, mean (± SD), kg/m^2^		32.9±6.2	28.6±4.6	<0.001
Grade of spondylolisthesis	no	80 (56%)	64 (60%)	ns
	Grade 1	56 (39%)	34 (32%)	
	Grade 2	8 (6%)	8 (8%)	
Height of iliac crest	Type I	62 (43%)	22 (20%)	<0.001
	Type II	42 (29%)	44 (42%)	
	Type III	40 (28%)	40 (38%)	
Position of screw heads	Neutral	23 (16%)	15 (14%)	ns
	LA-1 position	61 (42%)	44 (42%)	
	LA-2 position	60 (42%)	47 (44%)	
Distance of B to M, mean (± SD), cm		5.4±1.3	4.9±1.1	0.007
Distance of D to M, mean (± SD), cm		6.7±1.4	6.5±1.2	ns
Distance of C to M, mean (± SD), cm		11.4±1.6	9.6±1.7	<0.001
A-B length (depth), mean (± SD), cm		9.8±1.3	8.3±1.1	<0.001
A-D length (depth), mean (± SD), cm		10.7±1.4	9.5±1.2	<0.001
A-C length (depth), mean (± SD), cm		12.2±1.6	10.1±1.6	<0.001
Angle-1, mean (± SD), degree		30.3±9.9	24.4±8.5	<0.001
Angle-2, mean (± SD), degree		23.0±9.6	16.7±7.4	<0.001
Sagittal angle, mean (± SD), degree		11.2±6.1	13.2±7	ns

The result of measurements

In the measurements before the PTS surgery, the distance from B to M and the depth of the TFNI's trajectories were shorter and Angle-1 was narrower in the male group than the female group. Before and after the PTS surgery, the results of all measurements in females and in males were statistically different from each other except for the length of the C-M distance. In females, the medial needle entry point (NEP) (NEP-B), medial TFNI trajectory (A-B trajectory), and VA (Angle-1) before PTS surgery were statistically significantly different compared to post-PTS surgery (p=0.001). Similar data and statistical results were also found in males (p=0.001).

After PTS surgery, the length of the A-B trajectory increased by 11.2% (0.9 cm) in females and 11.4% (1.2 cm) in males. NEP-B was found to be positioned more laterally in males compared to females (1.3 cm vs 1.6 cm, respectively). In addition, it was found that the VA in the axial plan decreased by 24.1% in females and 31.6% in males. There was no significant difference between males and females in Angle-3 measurements (p=0.103). However, it was found that it was possible to perform the L5-TFNI procedure in all patients before and after PTS surgery.

The results for BMI

In both sexes, the results revealed that having a BMI above 30 kg/m² resulted in an increase in the trajectory of the L5-TFNI (Table 2). Interestingly, there was no statistical association between BMI and VA before or after PTS surgery (data not provided).

**Table 2:** Statistical data for spondylolisthesis, height of iliac crest, and position of screw heads. The table shows the statistical relationship between the eight measurements and the BMI, the grades of spondylolisthesis, the heights of the iliac crests, and the axial positions of the screw heads of the implants in females and males. One-way analysis of variance was used for comparison of groups and Tukey multiple comparison tests were used for comparison of subgroups. C, the most medial entry point of the needle where transforaminal nerve injection allowed by the iliac crest can be performed before posterior transpedicular stabilization surgery; M, midline; B, the most medial entry point of the needle where transforaminal nerve injection can be performed before transpedicular stabilization surgery; D, needle entry point on the skin surface for the trajectory of transforaminal nerve injection passing just lateral to the screw head after transpedicular stabilization surgery;  A, the target point for transforaminal nerve injection; BMI, body mass index; N, neutral position (≤5˚); LA-1 position, angled laterally between 5˚ and 15˚; LA-2 position, angled laterally ≥15. P<0.05 was considered statistically significant. The statistical significance levels were expressed as p<0.05, p<0.01, p<0.001. ns indicates p>0.05.

		C-M distance	B-M distance	D-M distance	A-C length	A-B length	A-D length	Angle-1	Angle-2
Female									
BMI (above 30 kg/m²)		<0.001	ns	0.042	<0.001	0.021	<0.001	ns	ns
Spondylolisthesis		ns	ns	ns	ns	ns	ns	ns	ns
Height of iliac crest	Total	0.001	ns	ns	<0.001	ns	ns	ns	ns
	Type I/II	ns	ns	ns	ns	ns	ns	ns	ns
	Type II/III	ns	ns	ns	0.006	ns	ns	ns	ns
	Type I/III	0.001	ns	ns	<0.001	ns	ns	ns	ns
Position of screw heads	Total	ns	ns	<0.001	ns	ns	<0.001	ns	0.001
	N/LA-1 position	ns	ns	ns	ns	ns	ns	ns	ns
	LA-1/2 position	ns	ns	0.002	ns	ns	0.004	ns	0.03
	N/LA-2 position	ns	ns	<0.001	ns	ns	0.001	ns	0.001
Male									
BMI (above 30 kg/m²)		<0.001	<0.001	<0.001	<0.001	<0.001	0.012	ns	ns
Spondylolisthesis		ns	ns	ns	ns	ns	ns	ns	ns
Height of iliac crest	Total	ns	ns	ns	<0.001	ns	ns	ns	ns
	Type I/II	ns	ns	ns	0.002	ns	ns	ns	Ns
	Type II/III	ns	ns	ns	ns	ns	ns	ns	ns
	Type I/III	0.03	ns	ns	<0.001	ns	ns	ns	ns
Position of screw heads	Total	ns	ns	<0.001	ns	ns	<0.001	ns	<0.001
	N/LA-1 position	ns	ns	0.004	ns	ns	ns	ns	ns
	LA1/2 position	ns	ns	0.01	ns	ns	<0.001	ns	0.005
	N/LA-2 position	ns	ns	<0.001	ns	ns	<0.001	ns	0.02

The results for spondylolisthesis

Spondylolisthesis was detected in 35% of all patients. But no statistical association was found between the grade of the spondylolisthesis and the eight measurements made.

The results for the height of the iliac crest

Type 1 was seen most frequently in females and Type 2 in males. There was a statistically significant relation between Type 3 and D-M distance (p=0.001 in males vs p=0.034 in females) and lengths of A-D trajectory (p = 0.001 in both sexes).

The results for the position of the SHs

The neutral position was less frequent in both sexes. SHs in the position LA-2 had a statistically significant association with the distance of C-M, the length of A-C trajectory, and the decrease of Angle-1 in both sexes. In males, it was found that the screw head had a statistically significant association with the increase in C-M length even in the LA-1 position (p=0.004).

## Discussion

Several studies have focused on defining the ideal transforaminal (TF) interventions to the L5-S1 level [12,13]. Ra et al. [12] studied the angles of fluoroscopy targeting the safe triangle. They found that the optimum VA was 30° oblique and 13.9° cephalocaudal tilt in females and 25° and 11° in males, respectively. Similar data were confirmed in the studies of Plastaras et al. [13]. In addition, they reported that 35% of cases required the use of the cephalad and 44% of the oblique angles, whereas the use of the caudal angle was not required in any of the cases. In our study, the sagittal angle was 11.2°±6.1° in females and 13.2°±7° in males, which is consistent with the above studies. On the other hand, our study mainly focused on how much VA changes in patients who underwent PTS surgery. We found that prior to PTS surgery, the VA of L5-TFNI was significantly narrower in male patients compared to female patients (24.4°±8.5° vs 30.3°±9.9°, p=0.001). Ra et al. [12] explained the difference by the wider pelvis in females and the more medial location of the iliac crest in males. But after PTS surgery, we found that the VA was narrowed in both sexes. The most important factors that made L5-TFNI difficult in those patients were the position of polyaxial SH and, rarely, the rods. We found that the SH in a position that was angled to the lateral more than 15˚ caused a decrease in the VA. According to the result, our recommendation is to lock the stabilization system while the polyaxial SHs are in a neutral position during the operation which can facilitate to perform L5-TFNI.

The L5-S1 transforaminal route is frequently used in transforaminal endoscopic discectomy (TFED). To reach L5-S1 foramen, some authors placed a pillow or pillows on the opposite side under the patient in the lateral position or they chose the approach 10-13 cm lateral to the midline after PTS surgery [3,4]. Choi et al. [10] investigated the height of the iliac crest in patients that underwent TFED. They reported that TFED can be performed if the upper limit of the iliac crest is below the middle level of the L5 pedicle in the sagittal plane, otherwise foraminoplasty should be performed. Foraminoplasty was required in 19% of their patients, and the height of the iliac crest was higher in those patients than those who did not. Patgaonkar et al. [11] defined a classification by evaluating the position of the iliac crest with respect to the L5 vertebra. They recommended that TFED is to be performed via transiliac approach in cases where the iliac crest remains above the L5 superior endplate line and the visualization of the lowest point of the spinous process of L5 is blocked by the pedicle when viewed from the iliac crest. In their study, 48.8% of patients who underwent TFED required a transiliac approach (27.7% of females and 62.9% of males). In our study, 32% of all patients had type 3 iliac crest, which was higher than the study of Choi et al. The rate of type 3 iliac crest was found as 27.3% in females and 37.7% in males in the present study. Our rates of type 3 iliac crest were similar to the incidence of type 3 iliac crest in females in the study by Patgaonkar et al., but the incidence in males was less than in their study.

We found that performing L5-TFNI was possible in all patients who have undergone PTS surgery. In our study, medial NEP was 6.7 cm and 6.5 cm in females and males, respectively, which provides the advantage of more medial intervention compared to the values in previous studies. In addition, before PTS surgery, lateral NEP was more laterally located in type 3 iliac crest than in types 1 and 2, and the trajectory was longer. The high iliac crest did not cause a decrease in VA after PTS surgery, which was inconsistent with the results in the above-mentioned studies. The explanation for these discrepancies may be that the thickness of the needle used for TFNI is thinner than the working cannula of the endoscope. 

A study has reported that TF injection could be easily performed unilaterally or bilaterally in patients with grade 1 or 2 spondylolisthesis who have not been operated [9]. In our study, the rate of cases with spondylolisthesis was 35%. In our study, no statistically significant relation was found indicating that spondylolisthesis made it difficult to perform TFNI before PTS surgery, and that result is consistent with the results of the previous study. In addition, the study revealed that it is possible to perform L5-TFNI after PTS surgery.

Some authors reported that BMI is an important factor in increasing trajectory length in TF interventions [16,17]. In a study, the mean distance of the TF route to the L5-S1 epidural area was 6.3 cm in the patients with a mean BMI of 19.9 kg/m^2^ [17]. In the same study, if the BMI value was below 18.5 kg/m^2^, the distance was found to be 6.18 cm, and if it is above 23 kg/m^2^, it was found to be 7.46 cm. In the other study having a limited number of pre-obese and class-1 obese patients, mean distances were 8.4 cm and 10 cm, respectively [17]. In both studies, a statistically significant relation was found between the length of the trajectory and BMI (p<0.001). In the current study, mean BMI and length of the trajectory before PTS surgery were 32.9±6.2 kg/m2 and 9.8±1.3 cm in females, and 28.6±4.6 kg/m2 and 8.3±1.1 cm in males, respectively. In addition, when both sexes were compared with each other and within the group, a statistically significant difference was found between BMI and trajectory length, consistent with the studies mentioned above.

## Conclusions

According to the study, the position of polyaxial SH and the rod of the implant, high iliac crest, and high BMI were important factors that made L5-TFNI difficult to be performed in patients who underwent PTS surgery. The most challenging of these three factors was the position of the SH angled laterally more than 15 degrees. Locking the stabilization system while the SHs are in the neutral position may facilitate possible L5-TFNI intervention in the future.
